# Comparison the Efficacy of Cefazolin plus Gentamicin with Cefazolin plus Ciprofloxacin in Management of Type –IIIA Open Fractures

**Published:** 2011-04-01

**Authors:** N Janmohammadi, M R Hasanjani Roshan

**Affiliations:** 1 Department of Orthopedics, Shahid Beheshti Hospital, Babol Medical University, Babol, Iran; 2 Infectious Diseases Research Center, Babol University of Medical Sciences, Babol, Iran

**Keywords:** Type IIIA open fracture, Antibiotic, Therapy, Cefazolin, Gentamicin, Ciprofloxacin

## Abstract

**Background:**

The optimal antibiotic regimen is still controversial in open fractures. The purpose of this study was to evaluate the efficacy of two different antibiotic regimens in management of type III-A open fractures.

**Methods:**

From January 2001 to January 2008, patients with type IIIA open fractures admitted in Shahid Beheshti Hospital Affiliated to Babol University of Medical Sciences were enrolled. Patients randomly received cefazolin plus gentamicin (group I) or cefazolin plus ciprofloxacin (group II). Both regimens were administered for 3 days. All patients were followed for 3 months. The efficacy of both regimens was compared.

**Results:**

One hundred-forty eight and 153 patients were treated in group I and II, respectively. The mean age of the patients treated in group I was 36.96±14.4 and in group II was 36.93±13.51 years. The rate of deep infection in group I was 5.4% and in group II was 6.5%. The efficacy of regimen I was 94.6% and regimen II was 93.5%.

**Conclusion:**

Cefazolin plus gentamicin, or cefazolin plus ciprofloxacin both can be successfully used for prevention of infection in type IIIA open fractures.

## Introduction

Wound and bone infections are frequently associated with open fractures of the extremities and may add significantly to the resulting morbidity. Antibiotics are effective in decreasing the incidence of infection in open fractures of the extremities compared to placebo. The administration of antibiotics as an adjunct to a comprehensive surgical management protocol including irrigation, surgical debridement and stabilization was shown to reduce the frequency of infection.[1]

The extent of the injury determines the appropriate antibiotic and the length of administration.[2] Inappropriate use of antibiotic promotes development of drug resistance, super-infections and increases the cost of the treatment.[3] The medical literature contains multiple reports comparing various antibiotic regimens in reducing infections and duration of therapy.[4][5][6][7][8][9][10] These studies were stratified for grade of open fracture according to Gustilo classification.[11][12] Generally in all types of open fractures, the antibiotic therapy should target both the gram–positive and the gram-negative pathogens contaminating the wound.[13] Zalavras et al. recommended a 3-day administration of first-generation cephalosporin and an aminoglycoside, supplemented with ampicillin or penicillin to cover anaerobes in farm or vascular injuries.[14]

Commonly used regimen consist of a first-generation cephalosporin (e.g., cefazolin), which is active against gram-positive organisms, combined with an aminoglycoside (e.g. gentamicin or tobramycin) which is active against gram-negative organisms. Substitutes for aminoglycosides include quinolones, aztreonam, third-generation cephalosporins, or other antibiotics that are effective against gram-negative organisms.[9][15]

Type-III open fractures are subdivided into IIIA, IIIB, and IIIC according to Gustilo et al. classification, based on the severity of open fractures.[12] To the best of our knowledge regarding to subdivision of type-III open fractures, there is no report indicating antibiotic therapy for specific subtype of type-III open fractures. The purpose of this study was to evaluate the efficacy of cefazolin plus gentamicin versus cefazolin plus ciprofloxacin in management of type -IIIA open fractures.

## Materials and Methods

From January 2001 to January 2008, 301 patients with grade IIIA open fractures (according to Gustilo et al. classification)[12] who attended Department of Orthopedics in Shahid Beheshty Hospital affiliated to Babol University of Medical Sciences entered the study. Exclusion criteria were patients younger than16 years, those with hypersensitivity to cehalosporins, flouroquinolones, renal impairment, open fractures involving short bones, diabetic and immune compromised patients, pregnant women, nursing mothers, and patients who were unable or not allowed to take oral medication within a 3 days of study period. Patients were randomly divided into two groups (Group I: 148 patients and group II: 153 patients). The study was approved by the Infectious Diseases Research Center Ethics Committee of the Babol University of Medical Sciences. All patients gave their written informed consent.

All fractures underwent timely irrigation, debridement and appropriate skeletal stabilization when indicated. Group I, received one gram cefazolin intravenously (IV) every 8 hours plus gentamicin (5 mg / kg/day) in three divided doses for three days. Group two received one gram cefazolin intravenously (IV) every 8 hours plus ciprofloxacin orally (500 mg, thrice daily) for the same duration. All patients were followed for 3 months. The rate of deep infection and the efficacy of both regimens in these two groups were determined. The data were analyzed by SPSS software (version 15, Chicago, IL, USA). Student t and Fisher Exact tests were used when appropriate. The rate of infection and the efficacy of both regimens were compared. A p value < 0.05 was considered significant.

## Results

One hundred and eight (73%) patients in group I and 107 (70%) in group II were male. The mean age of patients treated in group I and II was 36.96±14.41 and 36.93±13.51 years, respectively. Characteristics of all patients treated in both groups are shown in Table 1. There were not any significant difference between the two groups regarding gender and age.

The most involved extremity was lower limb [101 (68%) in group I and 98 (64%) in group II]. The most involved bone in upper limb in both groups was radius and ulna (11.5% and 9.1%, respectively). Tibia and fibula were the most involved bones in the lower limb, in the both groups (40.5% and 37.3%, respectively). There was not any significant difference between the two groups regarding the involved bone and extremity (Table 1). The rate of deep infection in group I was 5.4% and in group II was 6.5%. The efficacy of regimen I was 94.6% and regimen II was 93.5% (p=0.68).

**Table 1:** Characteristics of patients in these two treated groups

**Group^a^**	**Group I No=148**	**Group II No=153**
Gender		
Male, no (%)	108 (73)	107 (70)
Female, no (%)	40 (27)	46 (30)
Mean age±SD	36.96±14.4	36.9±13.5
Upper limb fracture no (%)	32 (22)	37 (24)
Humerus, no (%)	6 (4.1)	9 (5.8)
Radius, no (%)	5 (3.4)	8 (5.2)
Ulnar, no (%)	4 (2.7)	6 (3.9)
Radius and ulnar, no (%)	17 (11.5)	14 (9.1)
Lower limb, no (%)	101 (68)	98 (64)
Femur, no (%)	22 (14.8)	20 (13)
Tibia, no (%)	19 (12.8)	21 (13.7)
Tibia and fibula, no (%)	60 (40.5)	57 (37.3)
Both extremity, no (%)	15 (10)	18 (12)

^a^ There were no statistically differences between two groups with regard to all variables

## Discussion

In this study, we found no difference between the efficacy of two antibiotic regimens (cefazolin plus gentamicin with cefazolin plus ciprofloxacin) in management of type-IIIA open fractures (p= 0.679). A review of the medical literature strongly supports the use of antibiotic prophylaxis in management of open fractures, but there is no consensus on selection of antibiotic, mode of administration, and duration of therapy and so many protocols have been tried.[2][4][5][6][7][8][9][10]

Petzakis et al. performed a prospective randomized study comparing the infection rates when penicillin plus streptomycin, cephalothin, and placebo were used. The rate of infection with penicillin and streptomycin was 9.7%, cephalothin 2.3% and placebo 13.9%.[4] Petzakis et al. also retrospectively reviewed their experiences with various regimens and concluded that for severely contaminated wounds, broad spectrum antibiotics must be administered as soon as possible after injury and should be initiated and continued for no more than 72 hours.[9]

Benson et al. compared clindamycin with cefazolin and found no difference in infection rate with either regimen. They demonstrated that any antimicrobial agent with Staphylococcus aureus coverage is an adequate effective prophylaxis for open fractures.[16] Dellinger reported that patients with open fractures benefit from the use of an antibiotic against Staphylococcus aureus.[17] A prospective study performed in Nigeria showed a positive bacterial culture rate of more than 70% in open fractures, and Staphylococcus aureus as the commonest microbial isolate (37.5%). The antibiotic sensitivity pattern revealed high efficacies for pefloxacin, ciprofloxacin and ceftriaxone against the isolated microorganism.[18]

Johnson et al. revealed no statistically difference in the rate of infection in severe open tibial fractures of type II and III with the use of the first versus third generation cephalosporin.[6] Various studies also suggested that cephalosporin as prophylactic antibiotic of choice for open fracture.[2],[11],[19],[20] Cephalosporin and aminoglycosides are currently recommended for infection prophylaxis in high-energy open tibial fractures.[10] Bendar and Panikh used cefazolin in type I/II/IIIA and cefazolin plus gentamicin or tobramicin in Type-IIIB/IIIC open fractures of lower extremities caused by blunt trauma in adults and they reported deep infection rate of 4.9%.[8] Patzakis et al. compared the efficacy of ciprofluxacin with cefamendol plus gentomicin in types I , II, and III open fractures and found that single-agent antibiotic therapy with ciprofloxacin was effective in treatment of type-I and type-II open fracture wounds. They also recommended that ciprofloxacin or other fluoroquinolons alone could not be used for type-III wounds. They suggested that fluoroquinolons (ciprofloxacin, ofloxacin, fleroxacin, pefloxacin, norfloxacin) in combination with an aminoglycoside can be used for type-III wounds. The fluoroquinolones are broad-spectrum antibacterial coverage with activity against gram-positive and gram-negative bacteria. These agents have several other advantages compared with current recommended antibiotics, which include less frequent dosing, administration by either oral or parental routes, lack of need for serum level monitoring such as those required for cephalosporin or gentamicin, and lack of nephrotoxicity.[9] With regard to advantages of flouroquinolones and the result of the present study, ciprofloxacin may be used instead of an aminoglycoside in combination with a first-generation cephalosporin (such as cefazolin), in management of type IIIA open fractures. This avoids the potential toxicity associated with aminoglycosides.

The main weakness of our study is lack of wound culture and antibiotic susceptibility of the organisms before initiation of antibiotics. As the susceptibility of the isolated organisms may differ from different centers, further studies are required to confirm our findings. In summary, the result of this study shows that cefazolin plus gentamicin, or cefazolin plus ciprofloxacin, with high success rates, can be used for prevention of infection in type IIIA open fractures.
